# The Effects of Tea Polyphenols in Feed on the Immunity, Antioxidant Capacity, and Gut Microbiota of Weaned Goat Kids

**DOI:** 10.3390/ani15040467

**Published:** 2025-02-07

**Authors:** Yimei Xiao, Longcheng Chen, Yuewen Xu, Xiaolin He, Shangquan Gan, Fuquan Yin

**Affiliations:** 1 College of Coastal Agriculture Science, Guangdong Ocean University, Zhanjiang 524091, China; xiaoym007@163.com (Y.X.); 13763002239@163.com (L.C.); 2112104033@stu.gdou.edu.cn (Y.X.); 13590036152@163.com (X.H.); 2 The Key Laboratory of Animal Resources and Breed Innovation in Western Guangdong Province, Department of Animal Science, Guangdong Ocean University, Zhanjiang 524091, China

**Keywords:** tea polyphenols, weaned goat kids, immune function, antioxidant function, cecal short-chain fatty acids, cecal microbes

## Abstract

In this present study, we aimed to investigate the effects of adding tea polyphenols to feed on the immunity, antioxidant capacity, and gut microbiota of weaned goat kids. Thirty weaned kids (Leizhou goats, average initial weight of 9.32 ± 1.72 kg, 2 months old) were randomly divided into five groups with six kids in each group, with half being male and half being female. The control (CON) group was fed the basal diet, and the four other groups were supplemented with 2, 4, or 6 g/kg tea polyphenols or 50 mg/kg chlortetracycline in the basal diet (denoted as the T1, T2, T3, and CTC groups, respectively). The results showed that compared to the CON and CTC groups, adding 4 or 6 g/kg tea polyphenols could increase the expression levels of serum antioxidant enzymes and intestinal antioxidant genes in the kids. It also increased the expression of *Nrf2* and *IL-10* in the intestine, while reducing the content and gene expression of cytokines (IL-1β, IL-6, IFN-γ, and TNF-α). Dietary supplementation with 4 or 6 g/kg tea polyphenols reduced the expression levels of *TLR4*, *MyD88*, and *NFκB* in intestinal tissue, activated intestinal protective mechanisms, and enhanced the immune defense of the intestinal epithelium. Compared to the CTC group, feeding tea polyphenols significantly increased the Simpson indices. However, adding 4 g/kg tea polyphenols significantly increased the relative abundance of *Verrucomicrobiota*, *Candidatus Soleaferrea*, the *Christensenellaceae R-7 group*, and *Prevotella*, as well as the acetic acid content in the cecum of the kids (*p* < 0.05). Overall, the results indicate that dietary supplementation with 4 g/kg of tea polyphenols can effectively maintain the homeostasis of the gut microbiota and enhance the anti-inflammatory and antioxidant capabilities of weaned kids.

## Simple Summary

Weaning stress induces oxidative stress, which exerts a detrimental influence on the growth and intestinal health of goat kids. Antibiotics are effective in alleviating weaning stress. However, the concerns associated with antibiotic resistance and residual side effects have motivated researchers to seek alternative strategies to address weaning stress. Tea polyphenols, as natural plant extracts, have certain biological activities such as antioxidant, anti-tumor, antibacterial, and anti-inflammatory ones. In this study, dietary supplementation with 4 g/kg of tea polyphenols could effectively maintain gut microbiota homeostasis in weaned goats, as well as enhance antioxidant and immune functions. The potential mechanism might be regulating the production of NO by modulating *iNOS* mRNA expression and synthesis, thus regulating antioxidant capacity. Moreover, it can enhance the immune defense of the intestinal epithelium and reduces inflammatory damage by inhibiting the *TLR4/MyD88/NFκB* signaling pathway and cytokine-related gene expression. This work provides further insights into the beneficial effects of tea polyphenols in goat production.

## 1. Introduction

The intestine, as a key organ for nutrient digestion and absorption, is the largest immune organ in the body. Therefore, effective gastric and intestinal function is crucial for animal health, growth, and production performance [1]. However, when goat kids are weaned, factors such as underdeveloped intestines, changes in dietary structure, mother–infant separation, and external environmental changes can cause nutritional and psychological stress. It brings a series of stress reactions to the growth, physiology, and immunity of young animals, resulting in problems such as low weight, high mortality, and increased incidence rates [2]. Some studies have shown that weaning may lead to oxidative stress, thereby damaging the intestinal barrier [3], causing the disruption of intestinal immune function [4,5], the imbalance of microbial communities [6], and diarrhea [7]. Therefore, how to alleviate the negative effects of weaning stress is an important prerequisite for producing healthy livestock products. Antibiotics have been widely used to reduce weaning stress responses [8], such as chlortetracycline, oxytetracycline, gentamicin, etc., but the side effects of drug resistance and residue have led to a global ban on mass feeding. Therefore, developing natural plant extracts with anti-inflammatory and antioxidant properties to replace antibiotics in agricultural production has become a global research focus.

Tea is one of the most widely consumed beverages globally, containing various bioactive compounds beneficial to health [9]. Tea polyphenols are a general term for polyphenolic substances in tea. According to chemical structure, tea polyphenols can be classified into catechins, flavonoids, phenolic acids, and other small amounts of polyphenols (such as epigallocatechin gallate, flavonoids, and tannins) [10]. They can be digested and broken down into catechins and flavonoids by the gastrointestinal tract and then absorbed by the intestine. These decomposition products can maintain stable biological activity and exert certain antioxidant capacity, which is achieved through hydrogen atom transfer through hydroxyl structures or single electron transfer through electron structures similar to free radicals, thereby inhibiting free radical reactions, reducing cell or tissue damage, and preventing intestinal tract diseases [11,12,13]. According to reports, polyphenols and their derivatives can promote the expression of antioxidant enzymes by upregulating the genes of antioxidant response elements (AREs) [14], restore the body’s redox homeostasis, and prevent systemic or local inflammation. In addition, they can counteract inflammatory stimuli by stimulating the activation of pathways such as the *Nrf2*, *NFκB*, and downstream iNOS and COX-2 pathway [15]. This is attributed to the phenolic hydroxyl group. As natural plant chemicals, tea polyphenols can increase the number of beneficial bacteria and promote their adhesion to intestinal epithelial cells, competing for nutrients and space to inhibit the proliferation of harmful bacteria [16]. Meanwhile, short-chain fatty acids and bacteriocins synthesized by bacteria have the functions of eliminating pathogens, enhancing intestinal mucosal barrier function, and maintaining intestinal microbiota balance [17].

Our previous research demonstrated that dietary tea polyphenols improved the growth and stress response and reduced the intestinal permeability of kids, while CTC did not affect the final weight. We found that feeding tea polyphenols at concentrations of 4 or 6 g/kg could increase the activity of antioxidant enzymes in the intestines of weaned goat kids, enhance the immune capacity of blood samples, promote the integrity of the intestinal barrier, and reduce intestinal damage [18]. However, there is currently no evidence to explain the mechanism by which tea polyphenols alleviate symptoms. Therefore, this article explores the mechanism of the effects of tea polyphenols on the antioxidant and immune abilities of weaned goat kids, as well as their effects on short-chain fatty acids and cecal microbiota, aiming to provide a reference for the intestinal health of young ruminants.

## 2. Materials and Methods

The experimental protocol applied in this study followed the guidelines of the Animal Care and Use Committee of Guangdong Ocean University (authorization number SYXK-2018-0147, 2018).

### 2.1. Materials

The tea polyphenols used in this experiment were provided by Xi’an Best Biotechnology Co., Ltd. (Xi’an, China). Tea polyphenols were extracted from green tea and obtained in the form of a brown powder with a distinct tea aroma. The content of tea polyphenols was 98.1%, that of catechins was 86.6%, and that of epigallocatechin gallate was 54.2% (the chemical analysis of tea polyphenols was provided by the production company [19]).

### 2.2. Goat Kids and Experimental Protocol

Briefly, 30 weaned goats (Leizhou goats, average initial weight of 9.32 ± 1.72 kg, 2 months old) were randomly divided into 5 groups, with 6 goats in each group, with half being male and half being female. We adopted a gradual weaning method and dwindled the feeding frequency until the kids were successfully weaned. During weaning, 100–200 g of concentrated feed was supplied 2–3 times a day, without restrictions on drinking water and high-quality roughage (Table 1). Mothers were provided with a basic diet and do not receive dietary supplements for treatment. After the kids adapted for 7 days, the main experimental period of 45 days began immediately. The control group (named CON) was fed only a basic diet, while the additive groups were supplemented by adding 2, 4, or 6 g/kg tea polyphenols or 50 mg/kg chlortetracycline (premix; effective concentration: 15%; Catalog No.: 20181015; Jinhe Biotechnology Co., Ltd., Hohhot, China) to the basic diet and were named T1, T2, T3, and CTC, respectively. The tea polyphenols and chlortetracycline fed every day were evenly mixed into the concentrated feed three times a day: in the morning, at noon, and at night. During the experiment, the goat kids were fed the concentrate first and then the roughage (fresh materials), and enough clean drinking water was provided every day.

Before the experiment begins, the kid houses, feeding pens, metabolic cages, water troughs, and feed troughs were thoroughly cleaned and sterilized. After vaccination (Foot-and-Mouth Disease(FMD) vaccine, Peste Des Petits Ruminants Vaccine), deworming, and numbering, six goat kids were placed in each pen and fed according to the designed diet. During the feeding period, the feeding methods, experimental environment, and management mode of each group were the same. All experiments were designed by one-way random experiment. Daily teosinte was added into pellet feed by TMR, and the formulation of the basal diet (Table 1) was in accordance with the nutritional requirements of the Feeding standard of Goat, China [27].

### 2.3. Sample Collection

On the 45th day of the experiment, blood samples were collected from the jugular vein. The blood samples were centrifuged at 1500× *g* for 10 min at 4 °C, and the serum was collected and stored at −80 °C for further analysis.

The goat kids were fasted for 12 h prior to slaughter at the end of the trial. After euthanasia through electrocution and bloodletting, all kids (about 115 days old) were quickly dissected to collect tissue samples from the duodenum, jejunum, and ileum [18]. The tissue samples were washed three times with pre-cooled phosphate buffer and immediately transferred to liquid nitrogen. At the same time, the contents of the cecum were collected and frozen with liquid nitrogen for the analysis of microbial communities and detection of short-chain fatty acid concentrations.

### 2.4. Determination of Serum Antioxidant Indicators

The serum’s GSH-Px (Catalog No.: A005-1-2), CAT (Catalog No.: A007-1-1), T-SOD (Catalog No.: A001-3), MDA (Catalog No.: A003-1), and T-AOC (Catalog No.: A015-1-1) levels were measured using the ELISA assay kit, and the detection method was strictly in accordance with the kit instructions (Nanjing Jiancheng Bioengineering Institute, Nanjing, China). The detection instrument was a Synergy HTX Multifunctional enzyme marker (Bio Tek Instruments, Inc., Winooski, VT, USA).

### 2.5. Determination of Intestinal Immune Indicators

After the frozen intestinal mucosa samples were thawed on ice, the intestinal mucosa samples (0.5 g) were weighed and added to pre-cooled saline at a mass-to-volume ratio of 1:9 (g/mL) and ultrasonically pulverized to prepare tissue homogenates. After centrifugation at 3000× *g* at 4 °C for 15 min, the supernatant was collected and stored in a −80 °C refrigerator. IL-1β (Catalog No.: MM-175101), IL-6 (Catalog No.: MM-35226O1), IL-10 (Catalog No.: MM-009801), IFN-γ (Catalog No.: MM-009501), TNF-α (Catalog No.: MM-009601), iNOS (Catalog No.: MM-75337O1), and NO (Catalog No.: MM-1704O1) were determined using ELISA kits according to the instructions of the kits (Jiangsu Meimian Industrial Co. Ltd., Yancheng, China).

### 2.6. RT-qPCR Analysis

The total RNA extraction (Catalog No.: RC112), reverse transcription to cDNA (Catalog No.: R323), and real-time quantitative polymerase chain reaction analysis employed the methods described by Xu et al. [18]. Primers were designed according to the mRNA sequence of goat target genes on the NCBI official website (Table 2) and then passed on to Shenggong Biotechnology Co., Ltd. (Shanghai, China), for synthesis. According to the instructions of the ChamQ Universal SYBR qPCR Master Mix (Catalog No.: Q711, Vazyme Biotech Co., Ltd., Nanjing, China), the relative mRNA expression of genes was determined using a real-time fluorescence quantitative PCR instrument (Bio-Rad Laboratories Co., Ltd., Shanghai, China) and the 2^−ΔΔCt^ method was used for calculation.

### 2.7. Determination of Short-Chain Fatty Acids

The cecum contents and ultrapure water were evenly mixed in a ratio of 1:9 and then centrifuged at 12,000× *g* for 10 min. The supernatant was added to a solution of phosphoric acid and ethanol, and further centrifuged at 12,000× *g* for 10 min. After being filtered through a 0.22 μm filter membrane, short-chain fatty acids were determined with a gas chromatograph (Trace 1310 and ISQLT, Thermo, Waltham, MA, USA), and the measurement procedure was in accordance with that described by Guo et al. [7].

### 2.8. DNA Extraction

The total DNA was extracted from the cecal contents according to Yang et al. [28]. After estimating the concentration and quality of DNA with 1% agarose gel, the sample was diluted to 1 ng/μL with sterile water.

### 2.9. 16S rRNA-Based Sequencing Analysis of Cecal Contents

Following the method of Yang et al. [28], primers were designed based on conservative regions, and sequencing adapters were added to the ends of the primers for PCR amplification. The products were purified, quantified, and homogenized to form a sequencing library. The library that was constructed and passed quality inspection was sequenced using Illumina NovaSeq 6000 (Illumina, San Diego, CA, USA). The raw image data files obtained from high-throughput sequencing were converted into raw sequenced reads through base calling analysis, and the results were stored in the FASTQ file format, which included sequence information and quality information.

### 2.10. Statistical Analysis

Firstly, the raw reads obtained from sequencing were filtered using Trimmomatic v0.33 software. Then, cutadapt 1.9.1 software was used to identify and remove primer sequences, resulting in clean reads without primer sequences. Finally, the divisive amplicon denoising algorithm 2 (DADA2) method in QIIME2 2020.6 was used for denoising [29]. After concatenating the double-ended sequences and removing the chimeric sequences, the non-chimeric reads were obtained, which were further divided into feature analyses (OTUs, ASVs), diversity analysis, difference analysis, correlation analysis, and functional prediction analysis.

All the experimental data were collated using Excel 2019 to establish a database. Data were analyzed using SPSS 26.0 (SPSS Inc., Chicago, IL, USA) and presented as the mean ± standard error (SEM). The data were analyzed using the linear effect and quadratic effect, and the results were assessed using Tukey’s multiple comparison test with one-way analysis of variance (ANOVA). The column chart was made using GraphPad Prism 8. Statistical significance was established at *p* < 0.05.

## 3. Results

### 3.1. Effects of Tea Polyphenols on Serum Antioxidant Capacity of Weaned Goat Kids

The effect of dietary tea polyphenols on the serum antioxidant capacity of weaned goat kids is shown in Table 3. Compared to the CON group, the activity of CAT in the serum of weaned goat kids in the T2, T3, and CTC groups was significantly increased (*p* < 0.05), while the content of MDA in the serum of goat kids in the T2 and T3 groups was significantly decreased (*p* < 0.05). In addition, the activities of T-SOD and GSH-Px in the serum of goats in the T2 and CTC groups were significantly higher than those in other groups (*p* > 0.05), while the activity of T-AOC in the T2 group was the highest (*p* < 0.05). The results showed that adding 4 or 6 g/kg tea polyphenols to the diet had the effect of improving the serum antioxidant capacity of weaned goat kids.

### 3.2. The Effects of Tea Polyphenols on the Expression of Antioxidant Genes in the Intestines of Weaned Goat Kids

**Figure 1:**
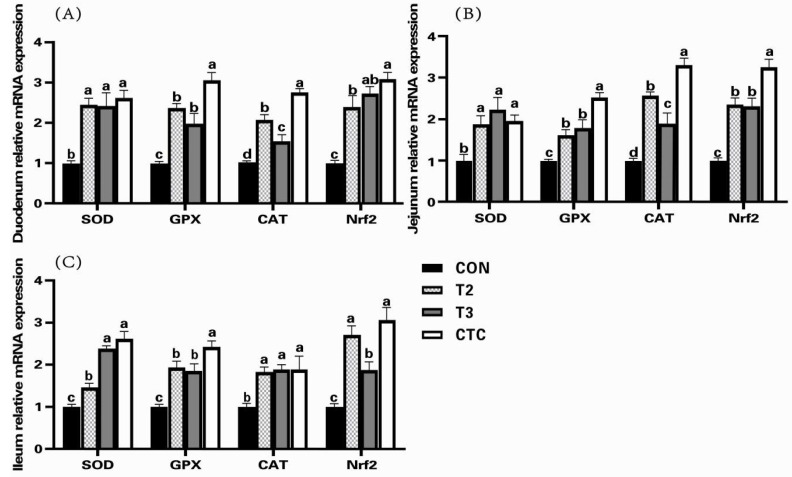
The effects of tea polyphenols on the expression of antioxidant genes in the intestines of weaned kids. CON: basal diet; T1: basal diet + 2 g/kg tea polyphenols; T2: basal diet + 4 g/kg tea polyphenols; T3: basal diet + 6 g/kg tea polyphenols; CTC: basal diet + 50 mg/kg chlortetracycline. (**A**–**C**): the relative gene expression of *SOD*, *GPX*, *CAT*, and *Nrf2* in the duodenum, jejunum, and ileum, respectively. The results are expressed as the mean ± SEM (*n* = 6). The mean values of ^a–d^ are significantly different (*p*  <  0.05).

To further evaluate the antioxidant capacity of dietary tea polyphenols, we compared the expression of antioxidant genes in the intestinal tract of weaned goat kids in the CON, T2, T3, and CTC groups. As evident from Figure 1, compared to the CON group, the relative expression levels of the *SOD*, *GPX*, *CAT*, and *Nrf2* genes were notably elevated in the intestines of weaned goat kids treated with tea polyphenols and antibiotics (*p* < 0.05). In the duodenum, there was no significant difference in the relative expression level of the *Nrf2* gene between the T3 and CTC groups (*p* > 0.05), while the CAT gene in the T2 group was significantly reduced compared to in other groups (*p* < 0.05). Simultaneously, compared to the tea polyphenol and CON groups, the *GPX* and *CAT* activities in the CTC group were significantly increased (*p* < 0.05). In the jejunum, compared to the other groups, the activities of the *GPX*, *CAT*, and *Nrf2* genes were notably increased in the CTC group (*p* < 0.05). However, the *CAT* gene activity in the T2 group surpassed that of the T3 group (*p* < 0.05). In the ileum, compared to the other groups, the *GPX* gene content was significantly elevated in the CTC group (*p* < 0.05), whereas the *Nrf2* gene content in the T3 group was notably reduced compared to both the CTC and T2 groups (*p* < 0.05).

### 3.3. Effects of Tea Polyphenols on Intestinal Immune Function in Weaned Goat Kids

To assess the anti-inflammatory properties of tea polyphenols, we measured cytokines indicative of inflammation in animals and investigated whether the supplementation of tea polyphenols could enhance the immune function of weaned goat kids’ intestines. The findings are presented in Table 4. Overall, the incorporation of tea polyphenols and aureomycin effectively decreased the concentrations of IL-1β, IL-6, TNF-α, and IFN-γ in the duodenum, jejunum, and ileum (*p* < 0.05), while it simultaneously elevated the concentrations of NO and IL-10 (*p* < 0.05). In the duodenum, dietary supplementation with tea polyphenols and chlortetracycline notably decreased the levels of IL-1β, IL-6, and TNF-α (*p* < 0.05) and significantly increased the levels of IL-10 and NO compared to the CON group (*p* < 0.05). Notably, the T2 group performed as well as the CTC group in terms of IL-1β expression. Additionally, the T3 and CTC groups exhibited significantly lower IFN-γ levels compared to other groups (*p* < 0.05), whereas their iNOS levels were notably higher (*p* < 0.05). Compared with the duodenum, similar expression patterns in these cytokines were observed in the jejunum and ileum, and the CTC group had the best anti-inflammatory effect. However, there was no significant difference in IL-6 levels between the T3 and CTC groups in the jejunum. Similarly, in the ileum, there was no significant difference in IL-6 and TNF-α levels between the two groups (*p* > 0.05).

### 3.4. The Effects of Tea Polyphenols on the Expression of Cytokine Genes in the Intestines of Weaned Goat Kids

**Figure 2:**
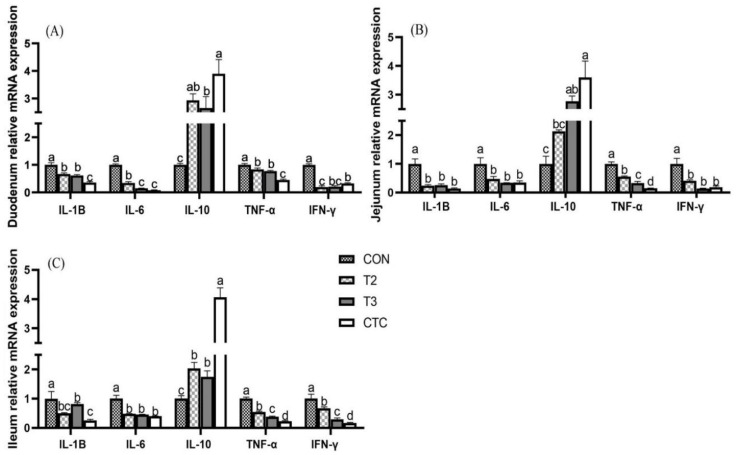
The effects of tea polyphenols on the expression of cytokine genes in the intestines of weaned kids. CON: basal diet; T1: basal diet + 2 g/kg tea polyphenols; T2: basal diet + 4 g/kg tea polyphenols; T3: basal diet + 6 g/kg tea polyphenols; CTC: basal diet + 50 mg/kg chlortetracycline. (**A**–**C**): the relative gene expressions of *IL-1β*, *IL-6*, *IL-10*, *IFN-γ*, and *TNF-α* in the duodenum, jejunum, and ileum, respectively. The results are expressed as the mean ± SEM (*n* = 6). The mean values of ^a–d^ are significantly different (*p* < 0.05).

Similarly, considering that tea polyphenols at doses of 4 or 6 g/kg had the best immune effects on goat kids, we selected the CON, T2, T3, and CTC groups to further compare the expressions of intestinal cytokine genes. As shown in Figure 2, compared to the CON group, supplementation with 4 g/kg and 6 g/kg of tea polyphenols, along with the antibiotic group, significantly reduced the gene expression levels of the cytokines *IL-1β*, *IL-6*, *IFN-γ*, and *TNF-α* in the duodenum, jejunum, and ileum of weaned goat kids (*p* < 0.05) while markedly increasing *IL-10* levels (*p* < 0.05). In the duodenum, the CTC group had the best immune response (*p* < 0.05), but the gene expression of *IFN-γ* in the T2 group was significantly lower than in the CTC group (*p* < 0.05). In the jejunum, there was no significant difference in the gene expressions of *IL-1β*, *IL-6*, and *IFN-γ* between the T2, T3, and CTC groups (*p* > 0.05), but *TNF-α* showed a gradually decreasing trend (*p* < 0.05). In the ileum, the gene expressions of *TNF-α* and *IFN-γ* in the CTC group were significantly lower than in other groups (*p* < 0.05), but the *IL-10* gene content was significantly higher (*p* < 0.05).

### 3.5. The Effects of Tea Polyphenols on the Expression of TLR4/NFκB Pathway-Related Genes and iNOS Gene Expression in the Intestines of Weaned Goat Kids

**Figure 3:**
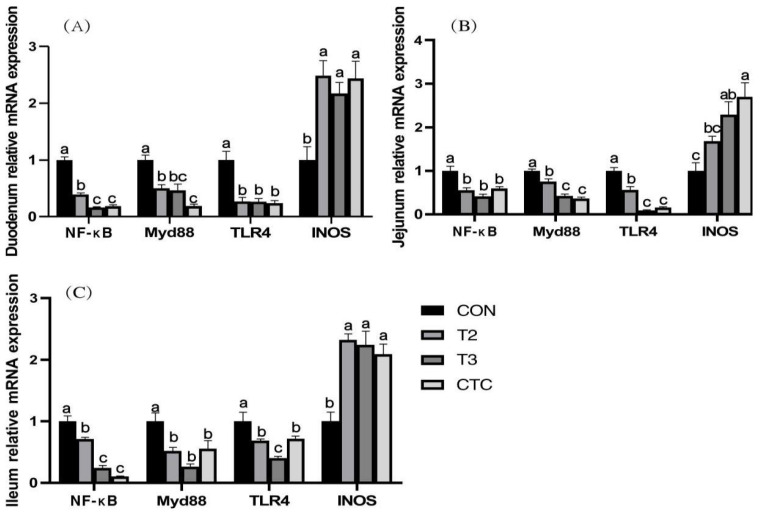
The effects of tea polyphenols on the expression of *TLR4/NFκB* pathway-related genes and *iNOS* gene expression in the intestines of weaned kids. CON: basal diet; T1: basal diet + 2 g/kg tea polyphenols; T2: basal diet + 4 g/kg tea polyphenols; T3: basal diet + 6 g/kg tea polyphenols; CTC: basal diet + 50 mg/kg chlortetracycline. (**A**–**C**): the relative gene expression of *NFκB*, *Myd88*, *TLR4* and *iNOS* in the duodenum, jejunum, and ileum, respectively. The results are expressed as the mean ± SEM (*n* = 6). The mean values of ^a–c^ are significantly different (*p* < 0.05).

According to Figure 3, overall, compared to the CON group, the gene content of NFκB, Myd88, and TLR4 in weaned goat kids fed with tea polyphenols and antibiotics significantly decreased (*p* < 0.05), while the expression level of the iNOS gene significantly increased (*p* < 0.05). In the duodenum, the content of the NFκB gene in the T3 and CTC groups was significantly lower than in other groups (*p* < 0.05). In the jejunum, the contents of the Myd88 and TLR4 genes were significantly lower than those in other groups (*p* < 0.05). Compared to the CON group, the expression levels of the NFκB gene in the T2, T3, and CTC groups were significantly decreased (*p* < 0.05), while the gene level of iNOS showed a stepwise increase (*p* < 0.05). In the ileum, the Myd88 gene content in the three additive groups was significantly lower than in the CON group (*p* < 0.05), and the iNOS level was significantly increased (*p* < 0.05). The expression levels of the NFκB gene in the T3 and CTC groups were significantly lower than in the other groups (*p* < 0.05), while the TLR4 level was the lowest in the T3 group(*p* < 0.05). The differences between the other groups were not significant (*p* > 0.05).

### 3.6. Effects of Tea Polyphenols on Short-Chain Fatty Acids in Cecum of Weaned Goat Kids

The content of short-chain fatty acids, such as acetic acid, propionic acid, isobutyric acid, and butyric acid, can affect intestinal barrier function and body metabolism. As shown in Table 5, the content of acetic acid in the cecum of the T2 group was significantly higher than in other groups (*p* < 0.05), while different concentrations of tea polyphenols and chlortetracycline had no significant effect on the content of propionic acid, isobutyric acid, butyric acid, and isovaleric acid (*p* > 0.05).

### 3.7. Effects of Tea Polyphenols on Composition of Cecal Bacteria in Weaned Goat Kids

By sequencing the bacterial 16 S rDNA V3 + V4 region, the microbiota of the cecal contents in the five groups of weaned goat kids were examined. High-throughput sequencing was conducted on three random cecum samples from each group, and the CON, T1, T2, T3, and CTC groups generated an average of 58,886, 55,578, 57,978, 67,672, and 52,331 non-chimeric reads (*n* = 3), respectively. These sequences were assigned to 31 phyla, 65 classes, 145 orders, 238 families, 430 genera, and 527 species based on a 97% similarity definition of the operational taxonomic unit (OTU).

**Figure 4:**
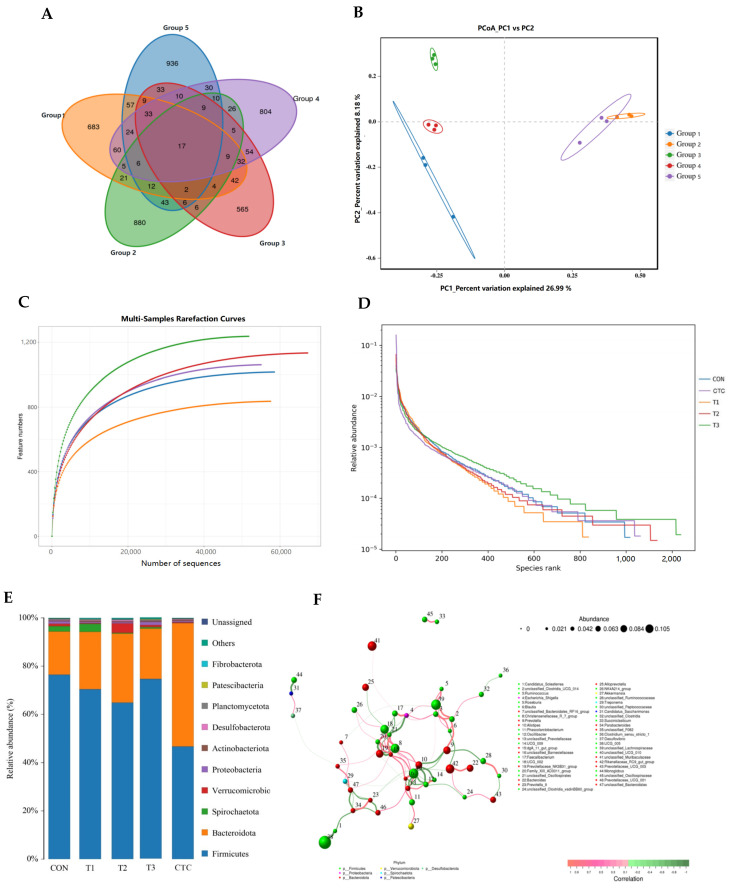
Effects of tea polyphenols on composition of cecal bacteria in weaned kids (*n* = 3). CON: basal diet; T1: basal diet + 2 g/kg tea polyphenols; T2: basal diet + 4 g/kg tea polyphenols; T3: basal diet + 6 g/kg tea polyphenols; CTC: basal diet + 50 mg/kg chlortetracycline. (**A**): Venn diagram. Group 1: CON; Group 2: CTC; Group 3: T1; Group 4: T2; Group 5: T3. Grouping settings are consistent with (**B**). (**B**): Principal co-ordinate (PCoA) analysis of OUTs. (**C**,**D**): Rarefaction curve (left) and rank abundance curve (right) of cecal microorganisms. (**E**): Species distribution bar chart. (**F**): Network diagram of species at genus level.

The Venn diagram displays the shared and unique microorganisms among different samples. The number of unique OTUs in the CTC group was 880, which was between the number of OTUs in the three treatment groups. In the three treatment groups given 2–6 g/kg tea polyphenols, the number of unique OTUs was 565, 804, and 936, respectively, showing an increasing trend. This was consistent with the overall trend of OTUs. These five groups had a total of 17 common OTUs, suggesting that different concentrations of tea polyphenols and chlortetracycline altered the existing microbial species in the intestines of weaned goat kids (Figure 4A).

When the number of valid sequences reaches 20,000, the microbial dilution curves of each group of samples tend to level off, suggesting that the detection was sufficient to cover all species and the sequencing quantity would not increase further (Figure 4C). Consequently, this implies that the microbial data are reliable. Figure 4D, the rank abundance curve, is a graphical representation of the feature abundance of each sample. The wider and flatter the curve is along the horizontal axis, the richer and more uniformly distributed the species composition is. The abscissal span increases with the increase in the tea polyphenol concentration, and this trend shows that the species abundance increased with the increase in dosage.

The Chao-1 index, Ace index, Shannon index, and Simpson index can be employed to measure the richness and diversity of microbial communities. The larger the Chao and ACE indices are, the higher the richness of microbial communities is. The larger the Shannon and Simpson indices are, the higher the diversity of microbial communities is. The alpha diversity analysis of gut microbiota is presented in Table 6. The Chao-1 index and Ace index of gut microbiota in each group do not exhibit significant changes (*p* > 0.05), suggesting that there was no remarkable alteration in the abundance of gut microbiota. However, the Simpson index of the cecum in the CTC group is significantly lower than that of other groups (*p* < 0.05). Meanwhile, the Shannon index of the CTC group is significantly lower than that of the CON and T3 groups (*p* < 0.05).

Principal coordinate 1 explains 26.99% of the variation among microbial colonies in all cecal contents, while principal coordinate 2 explains 8.18% (Figure 4B). From the figure, it can be seen that the CTC group and T3 group samples are the closest, indicating that there was not much difference in the microbial structural composition between them. The samples of the T1 and T2 groups are clearly separated from the control group, which indicates a significant difference in the composition of their microbial structures (*p* < 0.05). Thus, tea polyphenols and chlortetracycline affected the composition of cecal microbiota in weaned goat kids.

As shown in Figure 4E, tea polyphenols were found to alter the microbial community structure of the cecum in weaned goat kids. At the phylum level, Firmicutes and Bacteroidetes accounted for over 80% of the total microbiota, which may play a key role in maintaining the microbial environment. In this study (Table 7), compared to the CON group, the Firmicutes in the cecum of goat kids in the T2 and CTC groups were significantly reduced (*p* < 0.05), while the Bacteroidetes in the CTC group were significantly increased (*p* < 0.05). The Verrucomicrobota in the cecum of goat kids in the T2 group was significantly higher than that in the other groups (*p* < 0.05). However, the Proteobacteria levels in the cecum of goat kids in the high-dose T3 group were significantly higher than those in the T1, T2, and CTC groups (*p* < 0.05). The differences among the other groups were not significant (*p* > 0.05).

We further analyzed the top 10 genera with the highest species correlations (Figure 4F), and the results are shown in Table 8. Compared to the CON group, the abundance of Blautia in the cecal microbiota of goat kids in the T2 and T3 groups was significantly increased (*p* < 0.05), and the abundance of Prevotella in the T2 group was also significantly increased (*p* < 0.05). However, Candidatus_Soleaferrea and the Christensennellaceae_R_7_group in the cecum of goat kids in the CTC group were significantly lower than in other groups (*p* < 0.05). The differences among the other groups were not significant (*p* > 0.05).

## 4. Discussion

### 4.1. Effects of Tea Polyphenols on Antioxidant Capacity and iNOS in Weaned Goat Kids

When the body’s antioxidant system cannot clear the excess free radicals caused by weaning stress, redox homeostasis will be disrupted, resulting in the production of excessive reactive oxygen species (ROS) and damage to lipids, cell membranes, proteins, and nucleic acids, ultimately leading the occurrence of oxidative stress [30,31]. In the intestine, this manifests as the loss of intestinal function, impaired intestinal barrier function, and the induction of the intestinal inflammatory response [32,33]. Chen et al. [34] found that tea polyphenols fed to squabs could improve antioxidant enzyme activity in the gut, activate the *Nrf2-ARE* antioxidant pathway, induce the expression of *Nrf2* and downstream antioxidant enzyme-related genes (*SOD1*, *SOD2*, *CAT*, and *HO-1*), and improve the antioxidant capacity of squabs’ body and intestines. Malondialdehyde (MDA), as the main product of lipid peroxidation, can monitor the state of lipid oxidation. Wang et al. [35] found that adding natural green tea polyphenols to the diet could alleviate liver function damage in a D-galactose-induced oxidative aging model mice, increase GSH-Px and SOD content, reduce MDA expression, decrease oxidative stress, and maintain a balance between redox and inflammation in the body. Our research results indicate that 4 or 6 g/Kg tea polyphenols were found to enhance the antioxidant activity in the intestines of weaned goat kids, significantly reduce the MDA content, and boost the body’s antioxidant capacity.

Nrf2, an important nuclear transcription factor, can effectively regulate the activity of antioxidant enzymes in animal bodies by interacting with the antioxidant response element ARE protein. Most *Nrf2/ARE* signaling pathways not only inhibit reactive oxygen species (such as ROS and RNS), but they also induce the expression of a series of downstream protective target genes after activation (such as antioxidant genes, phase II detoxifying enzyme genes, molecular chaperone genes, etc.) [36]. Zhou et al. [37] found that adding tea polyphenol supplements to corn contaminated with low levels of fungal toxins as a diet for laying hens could not only alleviate the adverse reactions caused by toxin contamination but also increase the gene expression levels of *SOD3*, *Nrf2*, and *GSTs* in the liver, thereby enhancing the antioxidant capacity of laying hens. In addition, it has been reported that the overexpression of iNOS can protect liver damage by regulating oxidative stress. Tao et al. [38] demonstrated that tea polyphenol pretreatment weakened the down-regulation of iNOS in I/R-induced liver tissue in mice. This is similar to the results of this study, which showed an increase in NO and iNOS in goat kids in the T2 and T3 groups. In addition, the stimulating effect of tea polyphenols on intestinal NO content was consistent with *iNOS* mRNA expression in this experiment. But, in most other studies, Kudingcha can reduce the levels of NO and iNOS in oxidatively damaged mouse model, and catechins can also reduce the NO content in RAW 264.7 macrophages, inhibiting the production of ROS [39,40]. This may have been due to the increased levels of NO and iNOS in the intestines in this experiment, which also stimulated an increase in gene expression levels. In addition, some researchers suggest that elevated iNOS expression aids animals in adapting to hypoxic or stressful conditions [41]. Similarly, cell experiments have shown that the exposure of endothelial cells to NO or peroxynitrite donors can lead to an adaptive increase in glutathione (GSH) synthesis and the expression of *Nrf2* genes [42]. These findings suggest that active compounds in tea polyphenols regulate NO production by modulating *iNOS* mRNA expression and synthesis, thereby modulating the antioxidant capacity of weaned goat kids.

### 4.2. Effects of Tea Polyphenols on Expression of Cytokines and TLR4/NFκB Pathway-Related Genes in Kid Intestinal Cells

The immune function of the intestinal mucosa is mediated by immune cells and cytokines. Oxidative stress in animals not only causes direct cellular damage but also activates *NFκB* in the liver, leading to the release of pro-inflammatory cytokines. NFκB, a key transcription factor, plays an important role in immune and inflammatory responses, as it can stimulate the expression of different pro-inflammatory cytokines, including genes encoding cytokines and chemokines [43]. In addition, *NFκB* is activated by various stimuli such as oxidative stress, cytokines, bacterial or viral antigens, and oxidized low-density lipoprotein, leading to its secretion in large quantities. It participates in regulating various genes that are important for cellular responses, including inflammation, innate immunity, growth, and cell death [44]. Interleukin-1 (IL), as a key immune regulatory cytokine, plays a crucial role in the body’s defense system. The *TLR4/NFκB* signaling pathway, as an upstream pathway for inflammatory factors such as IL-1β, IL-6, IFN-γ, and TNF-α, is one of the important pathways in the inflammatory response. TLR4, as an important recognition receptor on the cell membrane surface, can bind to the adaptor protein MyD88 upon activation, thereby regulating the expression levels of downstream genes and other transcription factors in the NF-κB signaling pathway [45,46]. Research has shown that tea polyphenols can alleviate TBBPA-induced gill inflammation in carp by inhibiting the expression of the TLR4/*NFκB* signaling pathway, reducing the expression levels of the cytokines IL-1β, IL-6, and TNF-α mRNA, and thereby alleviating inflammation [47]. In addition, Zhao et al. [48] discovered that theaflavins isolated from Pu’er tea can boost the expression of *TLR2*, *TLR4*, and *MyD88*, thus activating downstream *NFκB* and initiating inflammatory responses, thereby enhancing the innate immunity and anti-inflammatory effects of RAW264.7 macrophages. These results are similar to those of our study, which show that goat kids experience weaning stress, and the addition of 4 or 6 g/kg tea polyphenols significantly reduced the expression of cytokine mRNA and *TLR4/NFκB* pathway-related genes. Therefore, tea polyphenols may enhance intestinal epithelial immune defense and reduce inflammatory damage by inhibiting the *TLR4/MyD88/NFκB* signaling pathway and cytokine-related gene expression.

### 4.3. Effects of Tea Polyphenols on Short-Chain Fatty Acids in Intestinal Tract of Weaned Goat Kids

Short-chain fatty acids (SCFAs) have received widespread attention due to their positive effects on health. SCFAs are defined as fatty acids with fewer than six carbon atoms, primarily including acetate, propionate, and butyrate, which collectively constitute over 95% of total SCFAs with a typical ratio of 60:20:20 [49]. Among them, acetic acid produced by colonic bacteria is transported to the liver, where it is used as an energy source and a substrate for synthesizing cholesterol and long-chain fatty acids. Propionate influences food intake and glucose homeostasis, serving as a substrate for hepatic gluconeogenesis. Butyrate supports intestinal barrier function and reduces the occurrence of intestinal inflammation. The production of other SCFAs (formic acid, valeric acid, and hexanoic acid) is relatively low [50,51,52]. Some studies have shown that feeding Liubao tea extract to diabetic mice can reverse the decrease in SCFA content in mouse feces, increasing propionate and butyrate concentrations, modulating microbiota composition, enhancing the abundance of beneficial bacteria, improving the intestinal barrier, promoting epithelial cell growth, and reducing systemic lipopolysaccharides (LPS) and inflammation [53]. In this experiment, goat kids fed 4 g/kg tea polyphenols exhibited significantly higher levels of acetic acid in their cecum compared to the other groups. This phenomenon may have been due to the fact that the vast majority of short-chain fatty acids are produced by the rumen in ruminants and then absorbed and utilized by rumen epithelial cells or transported to the liver for metabolism. Therefore, there was no significant change in butyric acid and propionic acid in this experiment. Zhuang et al. [54] found that fermented tea residue increased fecal acetate levels in heat-stressed fattening cattle, while there was no significant change in propionate and butyrate levels. The study also demonstrated that fermented tea residue promotes microbial fermentation in the large intestine, maintains health, and mitigates the adverse effects of heat stress, aligning with the results of this experiment. These findings highlight the potential of tea polyphenols to modulate SCFA levels, thereby contributing to gut health and systemic well-being.

### 4.4. Eeffect of Tea Polyphenols on Gut Microbiota of Weaned Goat Kids

The gut microbiota plays a crucial role in digestion, the immune system, metabolism, and overall health. Therefore, maintaining the dynamic balance of the gut microbiota is essential for maintaining overall health. The microbiota in the gut system consists of a complex community of anaerobic bacteria, fungi, archaea, protozoa, and viruses, distributed throughout the entire gastrointestinal tract [55]. When the microbiota is imbalanced, it can lead to the destruction of the intestinal barrier, increasing the susceptibility of the body to certain diseases. Only high levels of microbial diversity and richness can help maintain its resistance and stability after being stressed [56]. Zhao et al. [57] found that adding 300 mg/kg tea polyphenols to sturgeon feed significantly increased the Chao1 and Shannon index values, enhancing microbial diversity and richness while reducing the *Firmicutes-to-Bacteroidetes* (F/B) ratio. *Bacteroidetes*, a dominant intestinal phylum, interact with T cells to promote IL-10 secretion, providing protection against colitis. Conversely, *Firmicutes* are associated with impaired intestinal barrier integrity, and lipopolysaccharide (LPS) leakage. Therefore, a high F/B ratio is considered an important biomarker of gut microbiota dysbiosis. *Proteobacteria*, another phylum, includes pathogens such as *Helicobacter pylori* and *Escherichia coli*, which are linked to intestinal damage and inflammation [58]. In this study, dietary supplementation with 4 g/kg tea polyphenols significantly increased the Shannon and Simpson indices of the gut microbiota in weaned goat kids, thereby enhancing biodiversity and increasing the abundance of *Bacteroidetes* and *Verrucomicrobiota*, while reducing the abundance of *Proteobacteria* and *Firmicutes*. Li et al. [59] found that antibiotics can cause disruption in the gut microbiota in mice, and oral tea polyphenols can significantly alleviate the antibiotic-induced decrease in gut microbiota abundance and diversity and increase the relative abundance of probiotics such as *Blautia*, *Roseburia*, and *Prevotella*. This is similar to the results of this study, which show that the abundance of *Blautia* and *Prevotella* in the cecal microbiota of goat kids at 4 or 6 g/kg was significantly increased. However, *Candidatus_Soleaferrea* and the *Christensennellaceae_R_7_group* in the cecum of goat kids in the CTC group were significantly lower than in other groups, indicating that the long-term use of antibiotics can reduce beneficial bacteria in the intestine. Among them, *Blautia* produces butyric and acetic acids, contributing to anti-inflammatory effects by preventing pathogen colonization and upregulating T cells [60,61]. *Prevotella* participates in glucose metabolism and inhibits the action of *Bifidobacterium* [62,63]. The *Christensenellaceae_R_7_group* plays a crucial role in the degradation of carbohydrates and amino acids into acetate and ammonia, and its abundance is positively correlated with improvements in lamb growth performance and meat quality [64,65]. Overall, these research results indicate that the long-term use of antibiotics can lead to a decrease in beneficial bacteria in the intestines of weaned goat kids, while tea polyphenols can alleviate intestinal ecological imbalances by promoting beneficial bacteria and inhibiting harmful bacteria. This helps to enhance intestinal health, improves intestinal barrier integrity, prevents colitis, and provides a feasible antibiotic alternative for managing weaning stress in goat kids.

## 5. Conclusions

In summary, the findings of our study demonstrate that dietary supplementation with 4 g/kg of tea polyphenols can regulate the generation of NO by modulating the expression and synthesis of iNOS mRNA. Consequently, this action enhances the antioxidant and immune functions of the intestines in weaned goat kids. Furthermore, it can augment the immune defense of the intestinal epithelium and mitigate inflammatory damage by inhibiting the *TLR4/MyD88/NFκB* signaling pathway and the expression of cytokine-related genes. Additionally, adding 4 g/kg of tea polyphenols can effectively preserve the homeostasis of the gut microbiota. These results offer theoretical support for the application of tea polyphenols as feed additives to promote intestinal health in weaned goat kids.

## Figures and Tables

**Table 1:** Composition and nutrient level of basal diet (dry matter basis).

Ingredients (%)	Content
Pennisetum × sinese (Hay)	50.00
Cornmeal	29.00
Soybean meal	10.00
Wheat bran	7.50
NaCl	0.50
CaHPO_4_	0.50
Limestone	0.50
Premix ^1^	2.00
Analytical results	
Dry matter ([20], %)	90.80
Crude protein ([21], %)	14.69
Crude fat ([22], %)	2.84
Acid detergent fiber ([23], %)	26.23
Neutral detergent fiber ([23], %)	39.90
Ca ([24], %)	0.54
P ([25], %)	1.10
Crude ash ([26], %)	7.90
Metabolic energy (calculation, MJ/Kg)	10.43

^1^ The premix provided the following, per kg of diet: VA, 17,500 IU; VD6, 200 IU; VE, 50 IU; Cu, 20 mg; Fe, 75 mg; Se, 0.4 mg; Mn, 80 mg; Co, 0.3 mg; I, 1.2 mg; Zn, 40 mg.

**Table 2:** Real-time PCR primer sequences.

Gene	Primer Sequences (5′-3′)	GenBank Accession No.	Length (bp)
* CAT *	F:CACTCAGGTGCGGGATTTCT	XM_004016396.5	163
	R:CTGGATGCGGGAGCCATATT		
*INOS*	F:ACGGGGACGGTAAAGACATC	XM_013971952.2	210
	R:CCGGGGTCCTATGGTCAAAC		
*GPX1*	F:CAGTTTGGGCATCAGGAAAACG	XM_004018462.5	128
	R:GCCTTCTCGCCATTCACCTC		
*SOD1*	F:CCATCCACTTCGAGGCAAAG	NM_001285550.1	124
	R:GCACTGGTACAGCCTTGTGTA		
*Nrf2*	F:TCTGCTGTCAAGGGACATGGA	NM_001314327.1	212
	R:CGCCGGTCTCTTCATCTAGT		
*NFκB*	F:GAAGAGAAGGCGCTCACCAT	XM_018066509.1	107
	R:ATCACAGCCAAGTGGAGTGG		
*MYD88*	F:ACTCATTGAGAAGAGGTGCCG	XM_013973392.2	139
	R:CTTGATGGGGATCAGTCGCT		
*TNF-α*	F:TGCACTTCGGGGTAATCGG	NM_001024860.1	144
	R:CGCTGATGTTGGCTACAACG		
*TLR4*	F:GGGTGCGGAATGAACTGGTA	NM_001285574.1	158
	R:CTGGGACACCACGACAATCA		
*IL-1β*	F:AATGAGCCGAGAAGTGGTGT	XM_013967700.2	136
	R:CAGTGTCGGCGTATCACCTT		
*IL-10*	F:TACCCACTCTGGGGTCTTGT	XM_005690416.3	121
	R:CTGCCAAGCTCATTCACACG		
*IFN-γ*	F:AGATCCAGCGCAAAGCCATA	NM_001285682.1	110
	R:TCTCCGGCCTCGAAAGAGAT		
*GAPDH*	F:GATGCCCCCATGTTTGTGATG	XM_005680968.3	160
	R:CGTGGACAGTGGTCATAAGTC		
*IL-6*	F:ATCTGGGTTCAATCAGGCGAT	NM_001285640.1	247
	R:TGCGTTCTTTACCCACTCGT		

**Table 3:** Effects of tea polyphenols on serum antioxidant capacity of weaned goat kids.

Items	Groups					SEM	*p*-Value
	CON	T1	T2	T3	CTC		
MDA (nmol/mgprot)	2.18 ^a^	2.00 ^a^	1.07 ^b^	0.82 ^b^	1.89 ^a^	0.162	0.002
GSH-Px (U/gprot)	65.29 ^c^	65.12 ^c^	97.52 ^b^	66.01 ^c^	106.28 ^a^	4.908	<0.001
CAT (U/mgprot)	2.31 ^c^	2.59 ^bc^	2.94 ^a^	2.77 ^ab^	2.64 ^b^	0.066	0.005
T-SOD (U/mgprot)	117.17 ^b^	117.43 ^b^	164.15 ^a^	128.24 ^b^	164.81 ^a^	5.948	<0.001
T-AOC (mmol/gprot)	0.56 ^cd^	0.51 ^d^	0.87 ^a^	0.61 ^c^	0.79 ^b^	0.037	<0.001

CON: basal diet; T1: basal diet + 2 g/kg tea polyphenols; T2: basal diet + 4 g/kg tea polyphenols; T3: basal diet + 6 g/kg tea polyphenols; CTC: basal diet + 50 mg/kg chlortetracycline. MDA: malondialdehyde; GSH-pX: glutathione peroxidase; CAT: catalase; T-SOD: total superoxide dismutase; T-AOC: total antioxidant capacity. SEM: the standard error of the mean. ^a–d^ Values in the same row with different letters are significantly different (*p* < 0.05). Results are presented as the mean ± SEM (*n* = 6).

**Table 4:** Effects of tea polyphenols on Intestinal immune function in weaned goat kids.

Items	Groups					SEM	*p*-Value
	CON	T1	T2	T3	CTC		
Duodenum							
IL-1β (pg/mL)	75.10 ^a^	59.79 ^b^	54.73 ^c^	49.77 ^d^	43.75 ^e^	2.054	<0.001
IL-6 (pg/mL)	141.35 ^a^	126.30 ^b^	122.91 ^b^	104.18 ^c^	81.18 ^d^	3.937	<0.001
IL-10 (pg/mL)	43.64 ^d^	49.91 ^c^	52.24 ^c^	58.08 ^b^	65.22 ^a^	1.429	<0.001
TNF-α (pg/mL)	266.69 ^a^	213.88 ^b^	212.14 ^b^	200.75 ^b^	154.36 ^c^	6.923	<0.001
NO (μmol/L)	30.00 ^d^	33.59 ^c^	34.78 ^c^	37.57 ^b^	43.17 ^a^	0.869	<0.001
iNOS (pg/mL)	75.78 ^d^	79.98 ^cd^	82.96 ^c^	103.65 ^b^	110.26 ^a^	2.668	<0.001
IFN-γ (pg/mL)	569.04 ^a^	560.0216 ^a^	528.01 ^a^	427.94 ^b^	350.79 ^c^	16.936	<0.001
Jejunum							
IL-1β (pg/mL)	66.60 ^a^	62.55 ^a^	56.39 ^b^	48,84 ^c^	39.63 ^d^	1.858	<0.001
IL-6 (pg/mL)	138.85 ^a^	122.39 ^b^	117.66 ^b^	94.67 ^c^	86.69 ^c^	3.678	<0.001
IL-10 (pg/mL)	42.19 ^d^	48.13 ^c^	53.50 ^b^	56.03 ^b^	63.60 ^a^	1.450	<0.001
TNF-α (pg/mL)	284.46 ^a^	236.28 ^b^	227.80 ^b^	202.40 ^c^	167.86 ^d^	7.363	<0.001
NO (μmol/L)	29.25 ^d^	33.94 ^c^	34.77 ^c^	39.68 ^b^	43.31 ^a^	0.945	<0.001
iNOS (pg/mL)	75.44 ^d^	79.73 ^cd^	84.99 ^c^	95.46 ^b^	106.99 ^a^	2.229	<0.001
IFN-γ (pg/mL)	610.97 ^a^	543.99 ^b^	489.79 ^c^	422.83 ^d^	367.66 ^e^	16.964	<0.001
Ileum							
IL-1β (pg/mL)	74.59 ^a^	64.91 ^b^	56.55 ^c^	52.49 ^d^	41.83 ^e^	2.104	<0.001
IL-6 (pg/mL)	131.52 ^a^	123.40 ^a^	120.88 ^a^	98.14 ^b^	88.67 ^b^	3.446	<0.001
IL-10 (pg/mL)	45.29 ^c^	47.88 ^c^	49.79 ^c^	56.67 ^b^	65.39 ^a^	1.459	<0.001
TNF-α (pg/mL)	262.52 ^a^	250.28 ^ab^	229.75 ^b^	184.37 ^c^	167.63 ^c^	7.312	<0.001
NO (μmol/L)	31.11 ^c^	32.42 ^c^	35.22 ^b^	36.84 ^b^	43.06 ^a^	0.832	<0.001
iNOS (pg/mL)	68.669 ^e^	78.74 ^d^	91.25 ^c^	103.74 ^b^	111.23 ^a^	2.990	<0.001
IFN-γ (pg/mL)	633.22 ^a^	507.41 ^b^	455.66 ^c^	431.73 ^c^	341.65 ^d^	18.064	<0.001

CON: basal diet; T1: basal diet + 2 g/kg tea polyphenols; T2: basal diet + 4 g/kg tea polyphenols; T3: basal diet + 6 g/kg tea polyphenols; CTC: basal diet + 50 mg/kg chlortetracycline. IL-1β: interleukin-1β; IL-10: interleukin-10; IL-6: interleukin-6; TNF-α: tumor necrosis factor-α; IFN-γ: interferon-γ. SEM: the standard error of the mean. ^a–e^ Values in the same row with different letters are significantly different (*p* <  0.05). Results are presented as the mean  ±  SEM (*n* = 6).

**Table 5:** Effects of tea polyphenols on intestinal short-chain fatty acids in weaned goat kids.

Items	Groups					SEM	*p*-Value
	CON	T1	T2	T3	CTC		
Acetic Acid	448.80 ^b^	660.50 ^b^	1281.85 ^a^	612.88 ^b^	715.56 ^b^	87.844	0.005
Propionic Acid	420.67	394.61	703.58	648.02	412.95	47.695	0.071
Isobutyric Acid	151.50	163.23	158.032	158.31	168.62	5.492	0.927
Butyric Acid	234.52	345.35	358.19	321.52	259.99	28.82	0.647
Isovaleric Acid	154.089	161.87	155.287	163.34	168.53	4.827	0.910

CON: basal diet; T1: basal diet + 2 g/kg tea polyphenols; T2: basal diet + 4 g/kg tea polyphenols; T3: basal diet + 6 g/kg tea polyphenols; CTC: basal diet + 50 mg/kg chlortetracycline. SEM: the standard error of the mean. ^a,b^ Values in the same row with different letters are significantly different (*p* < 0.05). Results are presented as the mean ± SEM (*n* = 3).

**Table 6:** Effects of tea polyphenols on α-diversity index of caecum microorganisms in weaned goat kids.

Items	Groups					SEM	*p*-Value
	CON	T1	T2	T3	CTC		
ACE	1123.65	1145.74	1390.47	1258.08	1075.16	52.046	0.343
Chao-1	1112.59	1135.93	1384.40	1249.713	1067.87	52.204	0.339
Simpson	0.988 ^a^	0.986 ^a^	0.992 ^a^	0.991 ^a^	0.963 ^b^	0.003	0.015
Shannon	7.68 ^a^	7.69 ^ab^	8.27 ^ab^	8.17 ^a^	7.09 ^b^	0.140	0.021
Coverage	>99%						

CON: basal diet; T1: basal diet + 2 g/kg tea polyphenols; T2: basal diet + 4 g/kg tea polyphenols; T3: basal diet + 6 g/kg tea polyphenols; CTC: basal diet + 50 mg/kg chlortetracycline. SEM: the standard error of the mean. ^a,b^ Values in the same row with different letters are significantly different (*p* < 0.05). Results are presented as the mean ± SEM (*n* = 3).

**Table 7:** Effects of tea polyphenols on cecal flora structure of weaned goat kids (phylum level).

Items	Groups					SEM	*p*-Value
	CON	T1	T2	T3	CTC		
* Firmicutes *	69.12 ^a^	61.83 ^ab^	50.29 ^b^	65.36 ^a^	48.03 ^b^	2.587	0.006
* Bacteroidota *	28.22 ^b^	34.27 ^ab^	36.74 ^ab^	26.04 ^b^	42.87 ^a^	1.952	0.014
* Spirochaetota *	0.98	0.99	1.05	0.68	0.27	0.191	0.735
* Verrucomicrobiota *	1.89 ^b^	1.49 ^b^	3.68 ^a^	0.57 ^b^	0.73 ^b^	0.348	0.006
* Proteobacteria *	0.932 ^ab^	0.785 ^b^	0.57 ^b^	1.57 ^a^	0.43 ^b^	0.128	0.014
* Actinobacteriota *	0.51	0.29	0.27	0.26	0.435	0.059	0.64

CON: basal diet; T1: basal diet + 2 g/kg tea polyphenols; T2: basal diet + 4 g/kg tea polyphenols; T3: basal diet + 6 g/kg tea polyphenols; CTC: basal diet + 50 mg/kg chlortetracycline. SEM: the standard error of the mean. ^a,b^ Values in the same row with different letters are significantly different (*p* < 0.05). Results are presented as the mean ± SEM (*n* = 3).

**Table 8:** Effects of tea polyphenols on cecal flora structure of weaned goat kids (genus level).

Items	Groups					SEM	*p*-Value
	CON	T1	T2	T3	CTC		
* Alistipes *	2.00	3.71	2.92	1.99	1.22	0.377	0.284
* Blautia *	0.17 ^b^	0.21 ^b^	0.72 ^a^	0.68 ^a^	0.30 ^ab^	0.078	0.019
* Candidatus_Soleaferrea *	0.41 ^a^	0.47 ^a^	0.65 ^a^	0.42 ^a^	0.18 ^b^	0.045	0.002
* Christensenellaceae_R_7_group *	5.83 ^a^	7.31 ^a^	4.13 ^a^	5.10 ^a^	2.69 ^b^	0.531	0.035
* Unclassified_Bacteroidales_RF16_group *	0.25	0.05	0.67	0.48	0.33	0.087	0.199
* Ruminococcus *	1.10	1.12	1.19	1.36	0.28	0.187	0.452
* Escherichia_Shigella *	0.06	0.14	0.7	1.22	0.03	0.254	0.557
* Unclassified_Clostridia_UCG_014 *	1.52	0.58	1.42	1.29	0.87	0.171	0.414
* Roseburia *	0.38	0.52	0.36	0.91	0.418	0.109	0.136
* Prevotella *	1.11 ^b^	0.30 ^b^	5.85 ^a^	3.20 ^ab^	2.49 ^b^	0.611	0.01

CON: basal diet; T1: basal diet + 2 g/kg tea polyphenols; T2: basal diet + 4 g/kg tea polyphenols; T3: basal diet + 6 g/kg tea polyphenols; CTC: basal diet + 50 mg/kg chlortetracycline. SEM: the standard error of the mean. ^a,b^ Values in the same row with different letters are significantly different (*p* < 0.05). Results are presented as the mean ± SEM (*n* = 3).

## Data Availability

The data presented in this study are available on request from the corresponding author.
